# The spectrum of *TP53* mutations in Rwandan patients with gastric cancer

**DOI:** 10.1186/s41021-024-00302-y

**Published:** 2024-03-08

**Authors:** Augustin Nzitakera, Jean Bosco Surwumwe, Ella Larissa Ndoricyimpaye, Schifra Uwamungu, Delphine Uwamariya, Felix Manirakiza, Marie Claire Ndayisaba, Gervais Ntakirutimana, Benoit Seminega, Vincent Dusabejambo, Eric Rutaganda, Placide Kamali, François Ngabonziza, Rei Ishikawa, Belson Rugwizangoga, Yuji Iwashita, Hidetaka Yamada, Kimio Yoshimura, Haruhiko Sugimura, Kazuya Shinmura

**Affiliations:** 1 Department of Tumor Pathology, Hamamatsu University School of Medicine (HUSM), 1-20-1 Handayama, Higashi-Ku, Hamamatsu, Shizuoka 431-3192 Japan; 2 Department of Biomedical Laboratory Sciences, School of Health Sciences, College of Medicine and Health Sciences, University of Rwanda, P.O. Box 3286, Kigali, Rwanda; 3 Department of Pathology, University Teaching Hospital of Kigali, P.O. Box 655, Kigali, Rwanda; 4 Université Catholique de Louvain, Médecine Expérimentale, Brussels, 1348 Belgium; 5 Department of Pharmacology, Sahlgrenska Academy, University of Gothenburg, Gothenburg, SE-40530 Sweden; 6 Department of Pathology, School of Medicine and Pharmacy, College of Medicine and Health Sciences, University of Rwanda, P.O. Box 3286, Kigali, Rwanda; 7 Department of Internal Medicine, University Teaching Hospital of Kigali, P.O. Box 655, Kigali, Rwanda; 8 Department of Health Policy and Management, Keio University School of Medicine, Tokyo, 160-8582 Japan; 9 Sasaki Institute Sasaki Foundation, 2-2 Kanda Surugadai, Chiyoda-Ku, Tokyo, 101-0062 Japan

**Keywords:** *TP53*, Mutation spectrum, Mutation pattern, Genetic analysis, Gastric cancer, Rwanda, Africa

## Abstract

**Background:**

Gastric cancer is the sixth most frequently diagnosed cancer and third in causing cancer-related death globally. The most frequently mutated gene in human cancers is *TP53*, which plays a pivotal role in cancer initiation and progression. In Africa, particularly in Rwanda, data on *TP53* mutations are lacking. Therefore, this study intended to obtain *TP53* mutation status in Rwandan patients with gastric cancer.

**Results:**

Formalin-fixed paraffin-embedded tissue blocks of 95 Rwandan patients with histopathologically proven gastric carcinoma were obtained from the University Teaching Hospital of Kigali. After DNA extraction, all coding regions of the TP53 gene and the exon–intron boundary region of *TP53* were sequenced using the Sanger sequencing. Mutated *TP53* were observed in 24 (25.3%) of the 95 cases, and a total of 29 mutations were identified. These *TP53* mutations were distributed between exon 4 and 8 and most of them were missense mutations (19/29; 65.5%). Immunohistochemical analysis for TP53 revealed that most of the *TP53* missense mutations were associated with TP53 protein accumulation. Among the 29 mutations, one was novel (c.459_477delCGGCACCCGCGTCCGCGCC). This 19-bp deletion mutation in exon 5 caused the production of truncated TP53 protein (p.G154Wfs*10). Regarding the spectrum of *TP53* mutations, G:C > A:T at CpG sites was the most prevalent (10/29; 34.5%) and G:C > T:A was the second most prevalent (7/29; 24.1%). Interestingly, when the mutation spectrum of *TP53* was compared to three previous *TP53* mutational studies on non-Rwandan patients with gastric cancer, G:C > T:A mutations were significantly more frequent in this study than in our previous study (*p* = 0.013), the TCGA database (*p* = 0.017), and a previous study on patients from Hong Kong (*p* = 0.006). Even after correcting for false discovery, statistical significance was observed.

**Conclusions:**

Our results suggested that *TP53* G:C > T:A transversion mutation in Rwandan patients with gastric cancer is more frequent than in non-Rwandan patients with gastric cancer, indicating at an alternative etiological and carcinogenic progression of gastric cancer in Rwanda.

**Supplementary Information:**

The online version contains supplementary material available at 10.1186/s41021-024-00302-y.

## Introduction

According to Global Cancer Statistics 2020, gastric cancer is the sixth most frequently diagnosed cancer with 1,089,103 cases in 2020 and the third leading cause of cancer death worldwide with 768,793 deaths in 2020 [1]. Projections indicate that low- and middle-income countries will have ≥ 80% of the global cancer burden by 2030 [2]. Therefore, understanding the molecular characteristics of gene mutations associated with gastric cancer will be important to improve survival outcomes and minimize the incidence of cancer in these regions [3].

The tumor suppressor gene *TP53* remains one of the most mutated genes in human cancers and is important for cancer genesis and progression [4]. Gastric cancer is highly associated with *Helicobacter pylori (H. pylori)* infection, which causes various cellular abnormalities, including genomic instability by producing double-strand breaks in the host genome [5]. Normal functioning TP53 protects human genome integrity by preventing these damages [5]. Conversely, loss of function for TP53 caused by inactivating mutations is associated with gastric cancer initiation and its worst prognosis [5, 6]. Determining the mutation status of *TP53* can be a tool to predict the best treatment options, while mutant *TP53* itself can be a target for cancer therapy [7]. However, the mutation status of *TP53* and its spectrum have not been studied in Rwandan patients with gastric cancer. Dietary variation, environment, and genetic factors are thought to contribute to the differences observed in *TP53* mutation spectra [8]. Therefore it is important to find the *TP53* mutations, which are the most frequent in a given cancer type and geography [9].

The prevalence of a particular mutational pattern in a given type of cancer represents a distinct mutation mechanism in cancers [10]. For instance, C > T and C > G mutations at CpG sites are thought to be caused by the activity of the apolipoprotein B mRNA editing enzyme, catalytic polypeptide-like (APOBEC) enzymes and have been primarily associated with breast cancer [10] and exogenous factors, like nitroso compounds, known to be involved in the pathogenesis of gastric cancer can also exacerbate this mutational process [8]. The most prominent *TP53* mutation pattern in gastric cancer is G:C > A:T with a dominant feature of C > T at CpG sites [11]. At least 20% of G:C > A:T mutations take place at hypermutable CpG dinucleotides in all cancer types and are mostly associated with cytosine to uracil deamination [10]. Since this type of mutation is prevalent in cancers associated with chronic inflammation [12], it demonstrates the role of inflammation in enhancing the deamination process in gastric carcinogenesis [11]. The predominance of G:C > A:T transitions at dipyrimidine sites are known to be associated with nonmelanoma skin cancer and melanoma, including CC > TT tandem mutations, which are due to UV-light-induced C = C double bonds at adjacent pyrimidines [12, 13]. Another mutation pattern, which is associated with lung cancer and hepatocellular carcinoma, is the G:C > T:A transversion with dominant feature G > T. In lung cancer, individuals who are exposed to polycyclic aromatic hydrocarbons (PAHs) have approximately 30% of these types of mutations in *TP53* [12]. In geographic area with poor food storage, mycotoxin aflatoxin is a major contaminant and has been associated with high prevalence of hepatocellular carcinoma (HCC) with a transversion at codon 249 (p.R249S; G:C > T:A) [12]. Lung cancer ranks seventh and liver cancer ranks fifth in Rwanda [14]; however, molecular studies linking them with PAHs [15] and the mycotoxin aflatoxin [16], respectively, remain lacking. The G:C > T:A transversion, with C > A as a dominant feature, was first described in neuroblastomas and adrenocortical cancers [17]. However, it was recently described by whole genome sequencing in gastric cancer as part of patterns associated with environmental mutational processes [11].

For many years, much attention has been given to genomic analysis, especially in Europe, North America, and Asian countries like Japan and China [18–21]. In a study conducted in the United States of America, a significantly higher frequency of *TP53* mutations in patients of African American descent was identified [22]. However, the African continent continues to face healthcare inequities because genomic data generated outside of Africa do not represent the African population [23]. Until 2020, only 375 (0.016%) of total publications retrieved on PubMed globally were studies done on cancer in the African population [24]. There is a need to uncover genomic patterns that are specific to the African population to provide precise medical care. According to Globocan in 2020, gastric cancer was the fourth leading cancer in terms of incidence and mortality in Rwanda, after breast, cervical, and prostate [14]. However, the limited publications on cancer genetics and genomics focused on gene mutations in breast [25, 26], colorectal cancers [27], and DNA analysis of human papillomavirus in cervical cancer [28, 29]. Gastric cancer-related molecular pathology studies have yet to gain attention in Rwanda, and given the scarcity of genomic data in Rwanda in general, this study aimed at obtaining information on *TP53* mutation status in Rwandan patients with gastric cancer. During this study, *TP53* mutation spectra in our cases were analyzed and subsequently compared to the spectrum of non-Rwandan patients with gastric cancer. To our knowledge, this report on *TP53* mutations in Rwandan patients with gastric cancer is first of its kind.

## Materials and methods

### Patients and tissue samples

During the 2020 to 2022 study period, 255 Rwandan patients were prospectively received from the Endoscopy Service at the Department of Internal Medicine at the University Teaching Hospital of Kigali (CHUK) in Rwanda. Of 255 patients, 221 (86.6%) signed the consent form to participate and 101 of 221 (45.7%) were confirmed to have gastric cancer on histopathological examination. Of these 101 cases, 4 cases were excluded from this study due to insufficient tissue material for DNA extraction and two samples were excluded due to low quality of extracted DNA. The study therefore included 95 gastric cancer cases.

### Histopathological diagnosis

Microscopic examination of the biopsies was first performed at CHUK (Rwanda), and tissue slides were reviewed by pathologists at Hamamatsu University School of Medicine, Japan. Biopsy specimens whose diagnosis was confirmed as carcinoma were included in this study. The histopathological characteristics were determined in carcinoma samples based on both the Laurén’s classification [30] and World Health Organization (WHO) tumor classifications [31].

### *H. Pylori* status

To detect the presence or the absence of *H. pylori* in the gastric cancer biopsies, conventional polymerase chain reaction (PCR) analysis for the *ureC* gene, which is present in *H. pylori*, but not in humans, was used after slightly modifying the previous quantitative PCR method by Suzuki et al. [32]. The primer pair specific to *ureC* was composed of 5′-GCATGCAATTGAATAAAGCC-3′ (forward) and 5′-GCCGCTATAACGGATCAAAT-3′ (reverse) [32]. PCR technique included the first segment of initial denaturation at 95℃ (15 min), the second segment for 45 cycles of denaturation (30 s at 94℃), annealing (30 s at 60℃) and extension (1 min at 72℃), and the third segment of final extension (10 min at 72℃). PCR products were electrophoresed in 2% agarose gel for 30 min at 100 V. Gel was stained in ethidium bromide for 30 min and *ureC* gene bands were captured and visualized with an ATTO gel documentation system (ATTO corporation, Tokyo, Japan).

### TP53 gene sequencing

The genomic DNA extracted from formalin-fixed paraffin-embedded (FFPE) blocks was examined at the Department of Tumor Pathology of Hamamatsu University School of Medicine, Japan. DNA isolation was carried out using the QIAamp DNA FFPE Advanced UNG Kits (Qiagen GmbH, Hilden, Germany) and following manufacturer’s protocol. Direct Sanger sequencing using PCR products amplified by the primer sets for each exon was used for TP53 gene sequencing. The sequences of the PCR primers are shown in supplementary Table S1. Fragments covering exon 2–11, including the entire coding region, and boundary regions of the TP53 gene were amplified by PCR with HotStarTaq DNA polymerase (Qiagen, Valencia, CA, USA). The PCR products were purified with Exo-SAP-IT (Thermo Fisher Scientific, Waltham, MA, USA) and directly sequenced in two directions with a BigDye Terminator v3.1 Cycle sequencing Kit (Thermo Fisher Scientific). The sequencing reaction was performed initially at 96℃ for 1 min followed by 25 cycles at 96℃ for 10 s, 50℃ for 10 s, and 60℃ for 4 min. The sequencing reaction products were purified, and then analyzed in the ABI 3130xL Genetic Analyzer (Thermo Fisher Scientific). Cases with suspected insertion–deletion mutations were assessed using TA cloning as previously described [27, 33].

### Mutation detection and interpretation of mutations

UniproUGENE version 45 [34] and GENETYX® version 14.1.0 (Genetyx Corporation, Tokyo, Japan) were used to align ABI sequences to the *TP53* reference genomic sequences. A deletion variant was suspected when a stretch of multiple fluorescent signals was seen in the ABI sequence. Once a variant was confirmed for the second time in a different PCR experiment or TA cloning for deletion cases, the variant was annotated according to the Human Genome Variation Society recommendations (HGVS) and the Joint Consensus Recommendation of the American College of Medical Genetics and Genomics and the Association for Molecular Pathology [35, 36]. Next, variant details were checked in the ClinVar database [37], and if the details were available, this variant was determined to be a known variant. In cases where the variant details were missing in ClinVar, other databases like COSMIC [38] and the *TP53* Database platform (http://tp53.isb-cgc.org) were consulted for more details. A variant was considered novel when its details could not be found in ClinVar, COSMIC and the *TP53* Database. Novel variant description was done using Mutalyzer 2 [39], a tool designed to automatically apply the HGVS guidelines in order to describe a variant. To predict the effect of the novel variant on TP53 protein, MutationTaster2021 was used [40]. In this study, variants with < 1% of population-level minor allele frequency (MAF) in a database of 1000 genomes [20] and ExAC [41] were considered mutations. Finally, data regarding the flanking sequences of the mutated bases were generated after uploading the list of *TP53* mutations into the *TP53* Database website (https://tp53.isb-cgc.org/search_gene_by_var). For novel mutations that could not be found in the database, the sequences were manually compared against the reference genome (GRCh38) on the NCBI website (https://www.ncbi.nlm.nih.gov/) in order to identify the nitrogen bases positioned immediately before and after the mutated base in the 5′ to 3′ direction.

### Immunohistochemistry

TP53 protein expression in gastric carcinoma with *TP53* missense mutations was evaluated via immunohistochemistry. Tissue FFPE blocks were cut into 4-µm-thick sections. After deparaffinization, immunostaining was performed using an automatic immunohistochemical stainer, the HISTOSTAINER (Nichirei Bioscience, Tokyo, Japan) with Histofine Simple Stain MAX PO (Nichirei) as previously described [42]. A primary antibody for TP53 (Mouse monoclonal, clone DO-7; Dako, Glostrup, Denmark) was used, and 3,3′-diaminobenzidine (DAB) (Dako) was used as a chromogen. Nuclear counterstaining was performed using hematoxylin. The staining signal was visualized using a Leica DMD 108 microscope (Leica Biosystems, Wetzlar, Germany). Representative photomicrographs were captured using the same microscope.

### Collection of publicly available data

Somatic *TP53* mutations data of 440 stomach adenocarcinomas deposited in The Cancer Genome Atlas (TCGA) database (TCGA ID: STAD) and 100 stomach adenocarcinomas in patients from Hong Kong reported by Wang et al. [43], were utilized in this study. These data were collected from The cBio Cancer Genomics Portal (http://cbioportal.org), a web platform for exploring, visualizing and analyzing multidimensional cancer genomic data [44]. From this platform, mutation data were downloaded as files of tab-separated values and then they were converted into Excel files for summary and organization. To generate results on the mutation spectrum, the list of *TP53* mutations [Genomic Description (hg19)] of each data set was uploaded to the *TP53* Database website. Results were downloaded as comma separated values files and subsequently imported into an Excel spreadsheet for curation.

### Mutation signature analysis

First, we extracted single base substitution data from the list of all *TP53* mutations in Rwandan and non-Rwandan patients. Second, we categorized them into six substitution patterns: C > A, C > G, C > T, T > A, T > C, and T > G. Finally, 96 substitution types and sequence contexts were counted for each population as in previous reports [9, 11]. The Signal platform (https://signal.mutationalsignatures.com) was used to estimate the single base substitution (SBS) mutation signature for Rwandan and non-Rwandan patients [45].

### Statistical analysis

Statistical analysis was performed by using statistical product and service solutions version 29.0 software (SPSS Inc., Chicago, IL, USA). Fischer’s exact test was used to calculate the *p*, and results were considered statistically significant at *p* ≤ 0.05. To control the false discovery rate (FDR) within the comparisons, the Benjamini–Hochberg procedure (FDR correction) was applied [46] by using R software version 4.0.3 [47] and Package RVAideMemoire version 0.9-83-2 [48].

## Results

### Clinicopathological characteristics of Rwandan patients with gastric cancer analyzed for *TP53* mutation

Genomic DNA of 95 Rwandan patients with gastric carcinoma was extracted and used in this study. Clinicopathological profiles of the analyzed cases are shown in Table 1. The mean age ± standard deviation was 60.6 ± 13.7 years with ages ranging from 29 to 99 years; patients aged between 65 and 74 were slightly more frequent with 25 (26.3%). There were 51 females (53.7%) and 44 males (46.3%). Anatomically, the majority of tumor specimens (54; 56.8%) came from the antrum, followed by the fundus (12; 12.6%). According to Laurén’s classification, histopathologically mixed-type carcinomas were more frequent with 36 (37.9%) cases, followed by diffuse type with 24 (25.3%) cases. According to WHO classification, mixed-type was predominant with 36 (37.9%) cases followed by tubular, moderately differentiated in 16 (16.8%) cases. With regards to *H. pylori* status in gastric carcinoma lesions, it was detected in 7/95 (7.4%) cases through PCR analysis of the *H. pylori ureC* gene (supplementary Fig. S1), which is consistent with the previous result [49].

**Table 1:** Clinicopathological characteristics of Rwandan patients with gastric cancer (*n* = 95)

Characteristics	Number of cases	Percentage
**Age (y.o.)**		
Mean ± standard deviation		(60.6 ± 13.7)
Range		(29–99)
**Age group**		
< 45	15	15.8%
45–54	16	16.8%
55–64	23	24.2%
65–74	25	26.3%
75-	16	16.8%
**Sex**		
Female	51	53.7%
Male	44	46.3%
**Anatomic site of the tumor**		
Cardia	8	8.4%
Sub-cardia	3	3.2%
Fundus	12	12.6%
Corpus	3	3.2%
Corpus-antrum	2	2.1%
Antrum	54	56.8%
Antro-pyloric region	4	4.2%
Pylorus	9	9.5%
**Lauren’s classification**		
Intestinal	22	23.2%
Indeterminate	6	6.3%
Diffuse	24	25.3%
Mixed	36	37.9%
Not defined	7	7.4%
**WHO classification**		
Papillary	5	5.3%
Tubular, well-differentiated	1	1.1%
Tubular, moderately differentiated	16	16.8%
Tubular, poorly differentiated	6	6.3%
Poorly cohesive, signet-ring cell phenotype	7	7.4%
Poorly cohesive, other cell types	17	17.9%
Mixed	36	37.9%
Undifferentiated carcinoma	5	5.3%
Neuroendocrine carcinoma	2	2.1%

### *TP53* mutations identified in Rwandan patients with gastric cancer

**Fig. 1:**
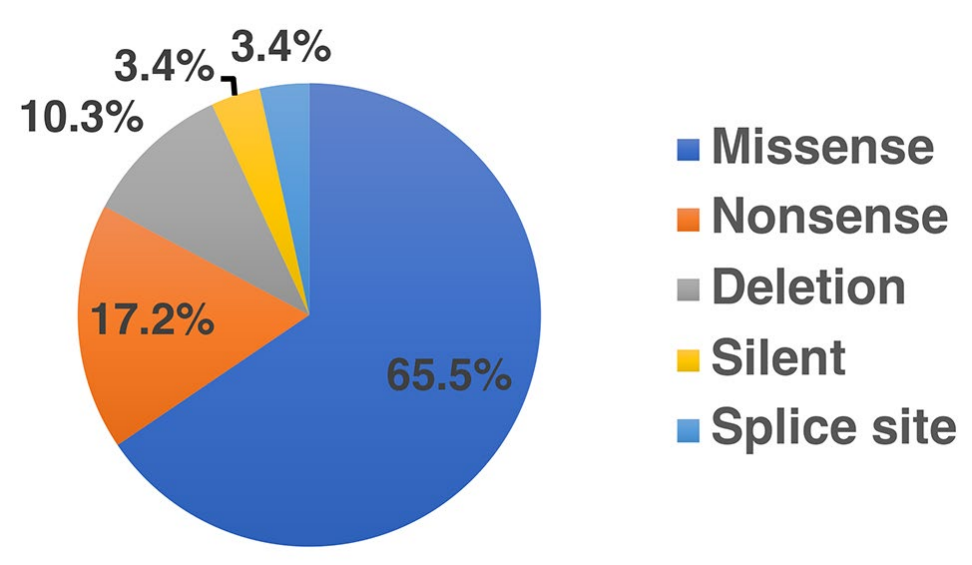
Types of *TP53* mutation in Rwandan patients with gastric cancer (*n* = 29). The pie graph shows different percentages of missense mutations (blue), nonsense mutations (orange), deletion mutations (gray), silent mutations (yellow), and splice-site mutations (light blue). Missense mutations were the most prevalent type with 65.5% of all mutations

**Fig. 2:**
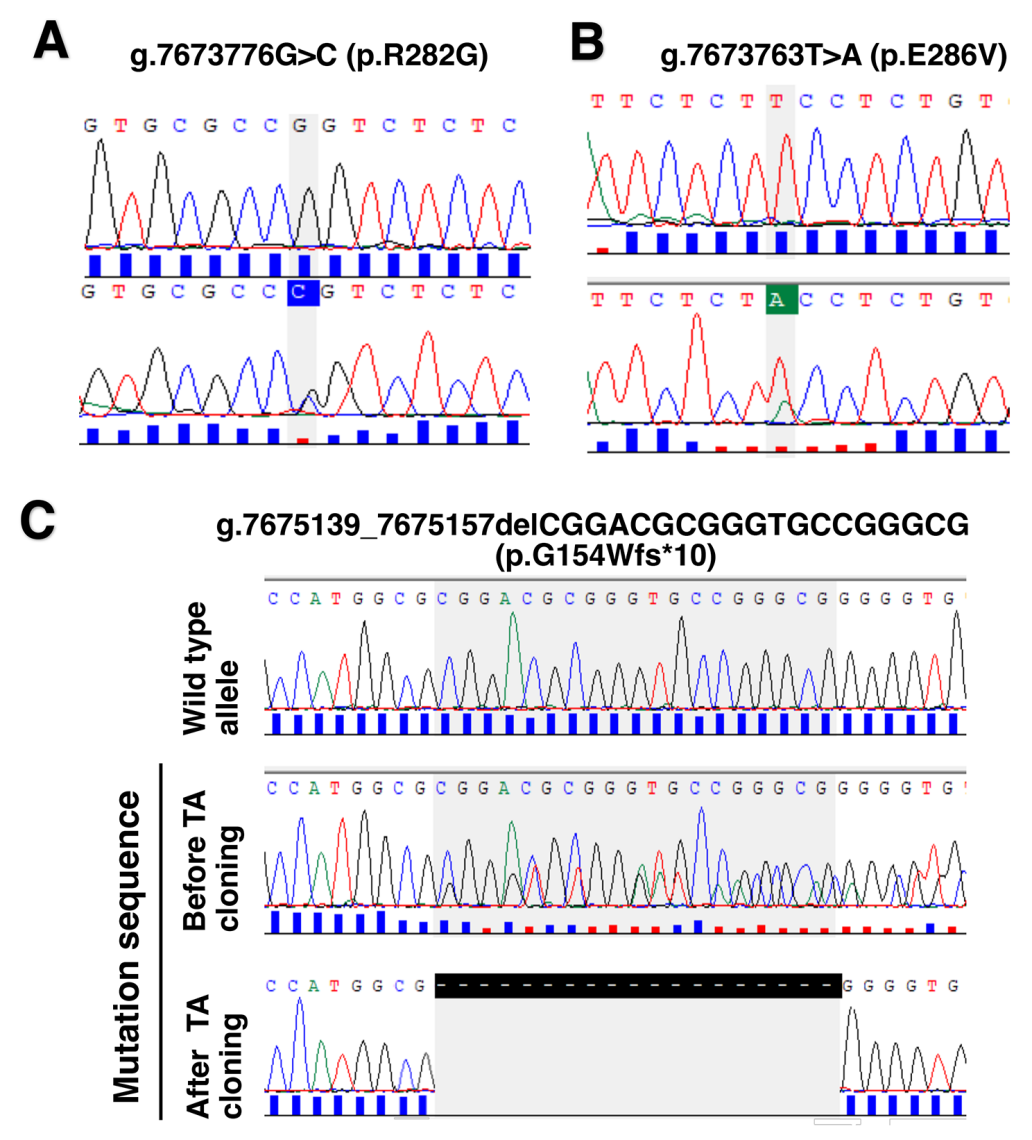
Representative electropherograms for the missense and novel *TP53* mutations in Rwandan patients with gastric cancer. **A**: p.R282G missense *TP53* mutation detected in case GC035. The lower electropherogram shows a g.7,673,776G>C (c.844C>G) *TP53* mutation corresponds to a missense mutation on codon 282, which is associated with p.R282G. The upper electropherogram is derived from a control case without *TP53* mutation. **B**: p.E286V missense *TP53* mutation detected in case GC089. The lower electropherogram shows a g.7,673,763T>A (c.857A>T) *TP53* mutation corresponds to a missense mutation on codon 286, which is associated with p.E286V. The upper electropherogram is derived from a control case without *TP53* mutation. **C**: A frameshift *TP53* mutation (g.7,675,139_7,675,157delCGGACGCGGGTGCCGGGCG corresponding to c.459_477delCGGCACCCGCGTCCGCGCC), which is associated with p.G154Wfs*10, was newly identified in this study. The middle and lower electropherograms are the results of gastric carcinoma containing this novel mutation in case GC021. In the middle electropherogram, mixed peaks are seen as result of the deletion of 19 bases. The lower electropherogram shows the *TP53* mutation sequence after TA subcloning. The shaded area represents 19 bases deleted. The upper electropherogram corresponds to the wild-type sequence (control case)

*TP53* mutations were observed in 24 (25.3%) of the 95 cases, with 29 total mutations (Table 2). These mutations (*n* = 29) were distributed between exon 4 and 8 with exon 5 having more mutations (12, 41.4%) followed by exon 6 (9; 32.0%) (supplementary Fig. S2). When these mutations (*n* = 29) were categorized by their effect on protein production, the majority were missense mutations (19; 65.5%), followed by nonsense mutations (5; 17.2%) and deletion type frameshift mutations (3; 10.3%) (Fig. 1). Representative sequencing results of missense mutations of the TP53 gene is shown in Fig. 2A and B [Fig. 2A shows g.7,673,776G>C (c.844C>G) mutation associated with p.R282G, while Fig. 2B shows the g.7,673,763T>A (c.857A>T) mutation associated with p.E286V]. Some mutations were detected more than once in different patients, these are c.637C>T (p.R213*) and c.527G>T (p.C176F), which appeared 3 times each, c.733G>A (p.G245S) and c.524G>A (p.R175H) appearing twice each (Table 2).

**Table 2:** List of *TP53* mutations identified in Rwandan gastric cancer patients

Sample ID	Exon /intron	Genomic description	Coding DNA description	Protein Description	Effect	dbSNP_ID
GC090	Exon 4	g.7,676,060G>T	c.309C>A	p.Y103*	Nonsense	NA
GC057	Exon 4	g.7,676,055C>A	c.314G>T	p.G105V	Missense	NA
GC060	Exon 5	g.7,675,206del	c.406del	p.Q136Nfs*34	Frameshift	NA
GC028	Exon 5	g.7,675,185C>T	c.427G>A	p.V143M	Missense	rs587782620
**GC021** ^**&**^	**Exon 5**	**g.7,675,139_7,675,157del**	**c.459_477del**	p.G154Wfs*10	**Frameshift**	**NA**
GC067	Exon 5	g.7,675,142A>G	c.470T>C	p.V157A	Missense	rs1131691023
GC019	Exon 5	g.7,675,139C>A	c.473G>T	p.R158L	Missense	rs587782144
GC014	Exon 5	g.7,675,138G>A	c.474C>T	p.Arg158=	Silent	rs139200646
GC053	Exon 5	g.7,675,094A>C	c.518T>G	p.V173G	Missense	rs1057519747
GC028	Exon 5	g.7,675,088C>T	c.524G>A	p.R175H	Missense	rs28934578
GC080	Exon 5	g.7,675,088C>T	c.524G>A	p.R175H	Missense	rs28934578
GC042	Exon 5	g.7,675,085C>A	c.527G>T	p.C176F	Missense	rs786202962
GC071	Exon 5	g.7,675,085C>A	c.527G>T	p.C176F	Missense	rs786202962
GC079	Exon 5	g.7,675,085C>A	c.527G>T	p.C176F	Missense	rs786202962
GC028	Exon 6	g.7,674,954G>A	c.577C>T	p.H193Y	Missense	rs876658468
GC075	Exon 6	g.7,674,950A>C	c.581T>G	p.L194R	Missense	rs1057519998
GC084	Exon 6	g.7,674,945G>A	c.586C>T	p.R196*	Nonsense	rs397516435
GC059	Exon 6	g.7,674,894G>A	c.637C>T	p.R213*	Nonsense	rs397516436
GC060	Exon 6	g.7,674,894G>A	c.637C>T	p.R213*	Nonsense	rs397516436
GC066	Exon 6	g.7,674,894G>A	c.637C>T	p.R213*	Nonsense	rs397516436
GC006	Exon 6	g.7,674,893C>A	c.638G>T	p.R213L	Missense	rs587778720
GC051	Exon 6	g.7,674,885C>T	c.646G>A	p.V216M	Missense	rs730882025
GC081	Exon 6	g.7,674,877del	c.654del	p.Y220Mfs*27	Frameshift	NA
GC038	Exon 7	g.7,674,230C>T	c.733G>A	p.G245S	Missense	rs28934575
GC067	Exon 7	g.7,674,230C>T	c.733G>A	p.G245S	Missense	rs28934575
GC014	Intron 7	g.7,674,180C>T	c.782+1G>A	p.?	Splice-site	NA
GC035	Exon 8	g.7,673,776G>C	c.844C>G	p.R282G	Missense	rs28934574
GC106	Exon 8	g.7,673,776G>A	c.844C>T	p.R282W	Missense	rs28934574
GC089	Exon 8	g.7,673,763T>A	c.857A>T	p.E286V	Missense	rs1057519985

& newly identified variant, ID: identification, NA: Not applicable, g.: genomic, c.: coding, p.: protein, dbSNP: The Single Nucleotide Polymorphism Database, rs: The reference single nucleotide polymorphism

When the *TP53* mutations found in this study were examined for novelty, 28 of 29 mutations were found registered in the ClinVar or COSMIC databases. The remaining mutation (g.7,675,139_7,675,157delCGGACGCGGGTGCCGGGCG corresponding to c.459_477delCGGCACCCGCGTCCGCGCC) was unavailable in the ClinVar, COSMIC, and *TP53* databases, considering this as a novel mutation. The newly identified mutation consists of the deletion of 19 bases between codon 459 and 477, resulting in the production of a short polypeptide of 10 amino acids in addition to the normally coded 154 amino acids, i.e., truncated protein of 164 amino acids (p.G154Wfs*10) (Fig. 2C: sequencing results of this frameshift mutation).

**Fig. 3:**
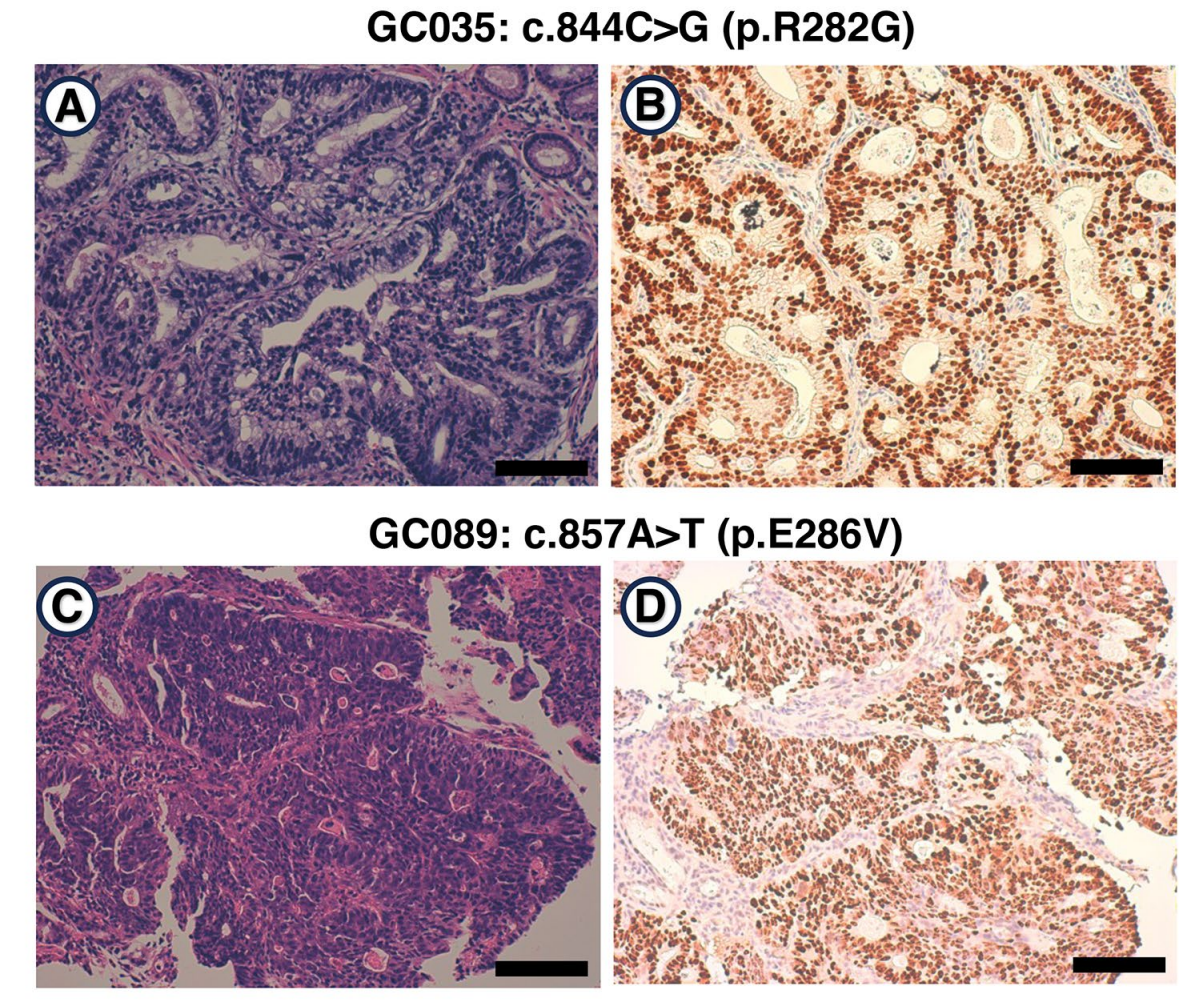
Representative photomicrographs for H&E staining and immunostaining for TP53 expression. **A**: The photomicrograph represents the H&E-stained slides showing *TP53* missense mutation-positive gastric carcinoma derived from case GC035. Note that the sequencing result of the *TP53* p.R282G missense mutation in this case is shown in Fig. 2A. **B**: The photomicrograph represents immunostaining for TP53 protein expression of gastric carcinoma containing a *TP53* p.R282G mutation in case GC035. **C**: The photomicrograph represents the H&E-stained slides showing *TP53* missense mutation-positive gastric carcinoma derived from case GC089. Note that the sequencing result of *TP53* p.E286V missense mutation in this case is shown in Fig. 2B. **D**: The photomicrograph represents immunostaining for TP53 protein expression of gastric carcinoma containing a *TP53* p.E286V mutation in the case GC089. Scale bar = 100 μm

Since *TP53* missense mutations are strongly associated with the TP53 protein accumulation in cellular nuclei [50, 51], we carried out immunohistochemical analysis for TP53 protein on 17 gastric cancer cases with *TP53* missense mutations. The results showed an abnormal accumulation of TP53 protein in 16 (94.1%) out of 17 cases (Fig. 3; immunohistochemical images of two representative cases are shown in Fig. 2A, B), suggesting the correctness of our TP53 gene sequencing.

No significant association was found between clinicopathological characteristics, including age, gender, anatomic site of carcinoma, histological classification, and *TP53* mutation (Table 3). Additionally, clinicopathological characteristics were not associated with the *TP53* mutation effects (missense mutation, frameshift mutation, nonsense mutation, silent mutation, and splice-site mutation) (supplementary Tables S2 and S3).

**Table 3:** Relationship between the clinicopathological characteristics and *TP53* mutation status in Rwandan patients with gastric cancer (*n* = 95)

Characteristics	Number of cases (percentage)		*p* value
	*TP53* mutant (*n* = 24)	*TP53* wild-type (*n* = 71)	
**Age (y.o.)**			0.276
Mean ± standard deviation	(63.3 ± 11.1)	(59.7 ± 14.5)	
Range	(40–80)	(29–99)	
**Age group**			0.190
< 45	2 (8.3)	13 (18.3)	
45–54	5 (20.8)	11 (15.5)	
55–64	3 (12.5)	20 (28.2)	
65–74	10 (41.7)	15 (21.1)	
75-	4 (16.7)	12 (16.9)	
**Sex**			0.956
Female	13 (54.2)	38 (53.5)	
Male	11 (45.8)	33 (46.5)	
**Anatomic site of the tumor**			0.620
Cardia	1 (4.2)	7 (9.9)	
Sub-cardia	0 (0.0)	3 (4.2)	
Fundus	3 (12.5)	9 (12.7)	
Corpus	0 (0.0)	3 (4.2)	
Corpus-antrum	0 (0.0)	2 (2.8)	
Antrum	15 (62.5)	39 (54.9)	
Antro-pyloric	1 (4.2)	3 (4.2)	
Pyloric	4 (16.7)	5 (7.0)	
**Laurén’s classification**			0.793
Intestinal	7 (29.2)	15 (21.1)	
Indeterminate	2 (8.3)	4 (5.6)	
Diffuse	4 (16.7)	20 (28.2)	
Mixed	9 (37.5)	27 (38)	
Not defined	2 (8.3)	5 (7.0)	
**WHO classification**			0.427
Papillary	0 (0)	5 (7.0)	
Tubular, well-differentiated	0 (0)	1 (1.4)	
Tubular, moderately differentiated	7 (29.2)	9 (12.7)	
Tubular, poorly differentiated	2 (8.3)	4 (5.6)	
Poorly cohesive, signet-ring cell phenotype	2 (8.3)	5 (7.0)	
Poorly cohesive, other cell types	2 (8.3)	15 (21.1)	
Mixed	9 (37.5)	27 (38)	
Undifferentiated carcinoma	2 (8.3)	3 (4.2)	
Neuroendocrine carcinoma	0 (0)	2 (2.8)	

### Comparison of the spectrum of *TP53* mutations in Rwandan patients with gastric cancer to previous *TP53* mutational studies on patients with gastric cancer

**Fig. 4:**
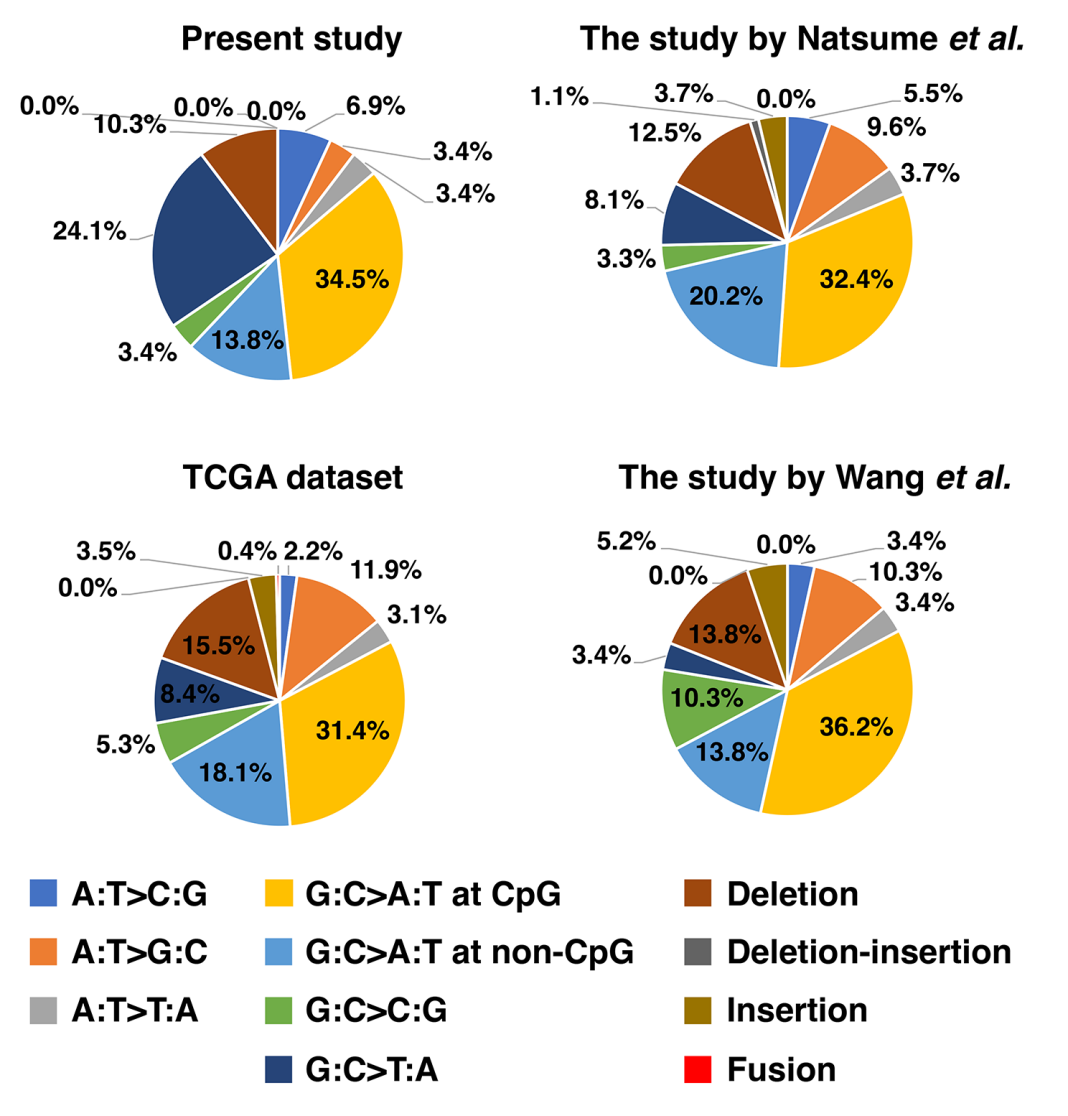
Spectrum of *TP53* mutations in Rwandan and non-Rwandan patients with gastric cancer. The data of Rwandan patients is from the present study (*n* = 29), while that of non-Rwandan patients is from the study by Natsume et al. (*n* = 272) [33], TCGA dataset (*n* = 226) [52], and the study by Wang et al. (*n* = 58) [43]. The pie chart illustrates the *TP53* mutation spectrum categorized into six types of single nucleotide substitutions as well as non-substitution mutations. The G:C > A:T transitions were subdivided into G:C > A:T at CpG sites and non-CpG sites. Each spectrum is shown in the pie graphs as follows: A:T > C:G (blue), A:T > G:C (orange), A:T > T:A (silver), G:C > A:T at CpG (yellow), G:C > A:T at non-CpG (light blue), G:C > C:G (green), G:C > T:A (dark blue), deletion (brown), deletion-insertion (grey), insertion (golden yellow), and fusion (red)

Next, we evaluated the spectrum (A:T > C:G, A:T > G:C, A:T > T:A, G:C > A:T, G:C > C:G, G:C > T:A, and deletion type) of *TP53* mutations detected in Rwandan patients with gastric cancer (Fig. 4 and supplementary Table S2). From a total number of 29 mutations identified in this study, 14 (48.3%) mutations were G:C > A:T (G > A or C > T). The G:C > A:T pattern was also divided based on their presence or absence at CpG sites. In this study, G:C > A:T at the CpG sites was most common (10; 34.5%), whereas G:C > A:T at non-CpG sites was 4 (13.8%). G:C > T:A was the second most frequent pattern (7; 24.1%) after G:C > A:T at CpG sites. The *TP53* mutation spectrum was not significantly associated with clinicopathological characteristics or area of residence in Rwanda (supplementary Table S4).

We then attempted to compare these *TP53* mutation spectrum of gastric cancer in Rwandan patients with previous *TP53* mutational studies on non-Rwandan patients with gastric cancer. The *TP53* mutation spectrum in our study (*n* = 29) was statistically compared with that of three previous studies, including our recent study by Natsume et al. (272 *TP53* mutations) [33], TCGA database (226 *TP53* mutations) [52], and the study by Wang et al. (58 *TP53* mutations) [43] (Table 4; Fig. 4). Age distribution and sex were not statistically significant between the present study and each of the three previous studies (supplementary Table S5). Among all forms of *TP53* mutations, the frequency of the G:C > T:A pattern was the only one significantly higher in Rwandan patients than in the studies on non-Rwandan patients by Natsume et al. (*p* = 0.013), the TCGA database (*p* = 0.017), and the study by Wang et al. (*p* = 0.006). In addition, even after FDR correction the statistical significances in the three previous studies were still observed using the Benjamini–Hochberg procedure with a maximum discovery rate of *d* = 0.05 (the adjusted *p* values: *p =* 0.034 for our study vs. study by Natsume et al., *p =* 0.034 for our study vs. TCGA dataset, and *p =* 0.033 for our study vs. study by Wang et al.). Conversely, no significant difference in the mutation effect on protein production was observed between Rwandan and non-Rwandan patients with gastric cancer (Table 4 and supplementary Fig. S3). These results suggest that G:C > T:A transversion mutation is more frequent in Rwandan patients with gastric cancer than non-Rwandan patients with gastric cancer.

**Table 4:** Frequency of the mutation spectrum of *TP53* in Rwandan patients with gastric cancer compared with the previous *TP53* mutational studies in non-Rwandan patients with gastric cancer

Mutation	Present study (*n* = 29)	Previous *TP53* mutational studies for gastric cancer patients					
		**Natsume** et al. [33]^***a***^ (*n* = 272)		**TCGA dataset** [52]^***b***^ (*n* = 226)		**Wang** et al. [43]^***c***^ (*n* = 58)	
	*n* (%)	*n* (%)	*p* ^d^	*n* (%)	*p* ^d^	*n* (%)	*p* ^d^
**Mutation spectrum**							
A:T > C:G	2 (6.9)	15 (5.5)	0.503	5 (2.2)	0.183	2 (3.4)	0.598
A:T > G:C	1 (3.4)	26 (9.6)	0.238	27 (11.9)	0.796	6 (10.3)	0.725
A:T > T:A	1 (3.4)	10 (3.7)	0.325	7 (3.1)	1	2 (3.4)	1
G:C > A:T at CpG	10 (34.5)	88 (32.4)	0.482	71 (31.4)	0.833	21 (36.2)	1
G:C > A:T at non-CpG	4 (13.8)	55 (20.2)	0.29	41 (18.1)	0.22	8 (13.8)	0.261
G:C > C:G	1 (3.4)	9 (3.3)	0.643	12 (5.3)	1	6 (10.3)	0.416
G:C > T:A	7 (24.1)	22 (8.1)	0.013**	19 (8.4)	0.017**	2 (3.4)	0.006**
deletion	3 (10.3)	34 (12.5)	0.51	35 (15.5)	0.588	8 (13.8)	0.745
deletion-insertion	NA	3 (1.1)	NA	NA	NA	NA	NA
insertion	NA	10 (3.7)	NA	8 (3.5)	NA	3 (5.2)	NA
fusion	NA	NA	NA	1 (0.4)	NA	NA	NA
**Mutation type**							
Missense	19 (65.5)	172 (63.2)	0.808	167 (73.9)	0.397	33 (56.9)	0.4395
Nonsense	5 (17.2)	42 (15.4)	0.8	NA	NA	10 (17.2)	1
Silent	1 (3.4)	10 (3.7)	0.95	NA	NA	NA	NA
Splice-site	1 (3.4)	1 (0.4)	0.052	19 (8.4)	0.42	4 (6.9)	0.515

NA: Not applicable

^a^Data collected from our previous work [33], the work consisted of 272 *TP53* mutations identified in 689 gastric cancer patients from China (*n* = 133), Eastern Europe (*n* = 288), and Japan (*n* = 268)

^b^The Cancer Genome Atlas (TCGA) data on *TP53* mutation in stomach adenocarcinoma accessed through cBioPortal for Cancer Genomics [52]. In the database, 213 stomach adenocarcinoma cases with *TP53* mutation are registered. The mutation-positive cases consisted of Black or African American (*n* = 8), Asians (*n* = 45), Whites (*n* = 135), and 25 cases without information on race. The total number of *TP53* mutations was 226

^c^Dataset from whole genome sequencing of 100 gastric cancers of patients from Hong Kong, in a study by the University of Hong Kong and Pfizer, accessible in cBioPortal for Cancer Genomics but also published in Nat Genet 2014 [43]. In this study 58 *TP53* mutations were seen in 55 patients with *TP53* mutation(s)

^d^The Fischer’s exact test between each study and the present study was performed

**indicate the significant difference (less than 0.05) in the *p*-value of Fischer’s exact test. The statistical significance was observed even after the Benjamin–Hochberg procedure with a maximum FDR of d = 0.05 for multiple comparisons [the adjusted p-values: 0.034 for this study vs. study by Natsume et al. [33], 0.034 for this study vs. TCGA study [52], and 0.033 for this study vs. study by Wang et al. [43]]

**Fig. 5:**
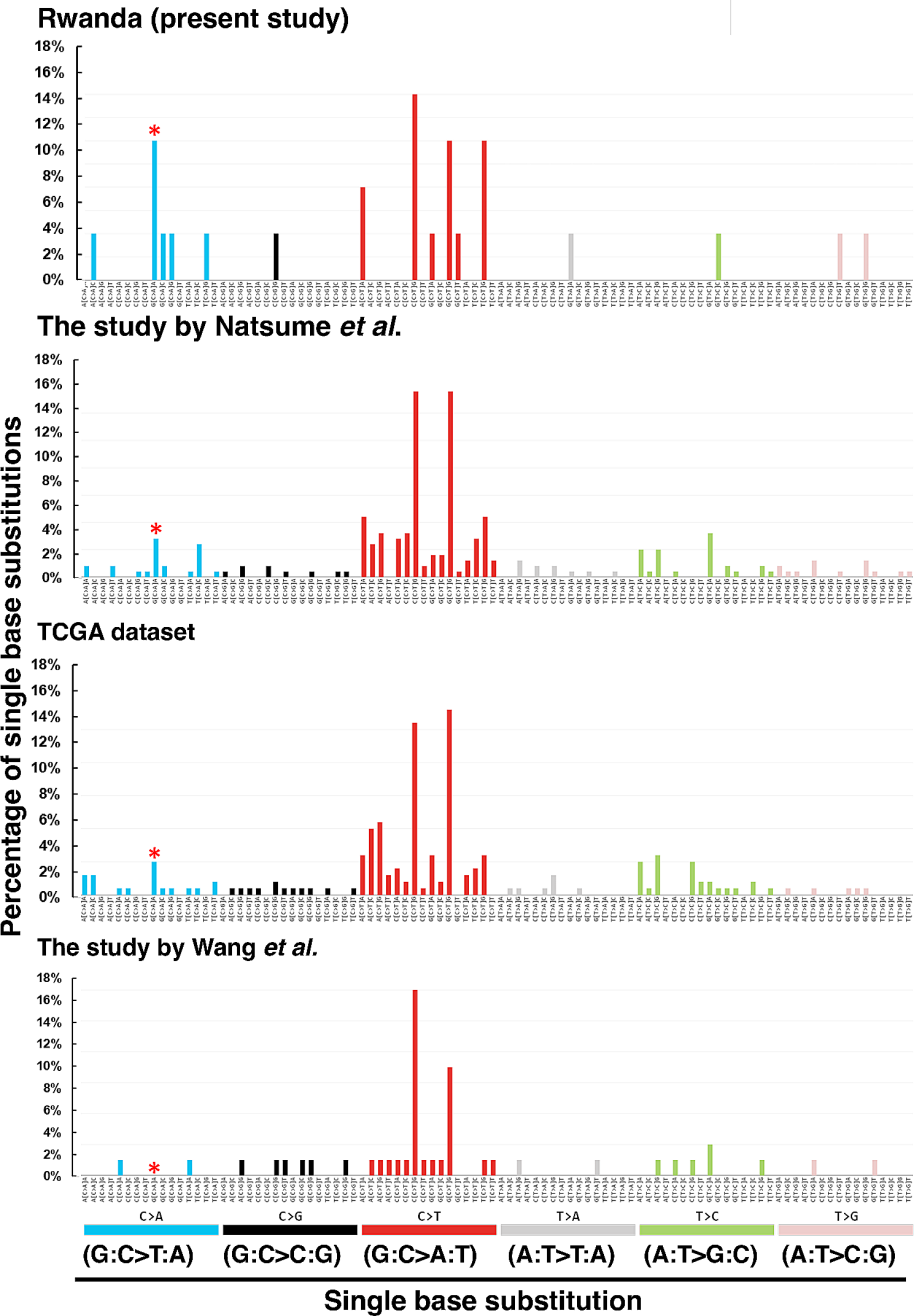
Landscape of *TP53* mutational substitutions in Rwandan and non-Rwandan patients with gastric cancer. The single base substitutions with their total number included Rwandan patients (this study [*n* = 26]), non-Rwandan patients (the study by Natsume et al. [*n* = 225] [33], TCGA dataset [*n* = 182] [52], and the study by Wang et al. [*n* = 47] [43]. The bar graphs illustrate different mutation counts based on the 96-substitution classification, and each bar represent a frequency of a particular mutation from the traditional six base substitutions namely, C > A (blue), C > G (black), C > T (red), T > A (light gray), T > C (green), and T > G (pink). Substitutions were investigated using their immediate sequencing context (the base immediately 5′ before the mutation and the base immediately 3′ after the mutation) to make 96 mutations. * represents G[C > A]A, the pattern most frequently found in G:C > T:A mutations of Rwandan patients with gastric cancer (11.5%) compared to non-Rwandan patients in the study by Natsume et al. (3.1%), the TCGA dataset (2.7%), and the study by Wang et al. (0.0%)

The estimated SBS mutation signatures were consistent with SBS1 in all four studies when the 96 mutation patterns were further analyzed (Fig. 5 and supplementary Table S6). Among C > A (G:C > T:A) mutation, the G[C > A]A mutation was the most frequently found (11.5%) in Rwandan patients with gastric cancer. On the contrary, this mutation demonstrated low frequencies of 3.1%, 2.7%, and 0.0% in these studies [33, 43, 52], respectively. Thus, G[C > A]A mutation may chiefly increase G:C > T:A mutations in Rwandan patients with gastric cancer.

### Possible involvement of APOBEC enzymes on *TP53* mutations in Rwandan gastric cancer

Since four of the APOBEC members (APOBEC3A, APOBEC3B, APOBEC3C, and APOBEC1) favor C residues immediately flanked by T at its 5′ side and APOBEC3G favors C immediately flanked by C at its 5′ side [53], flanking sequence context in G:C > A:T transitions, which is the most prevalent among the seven types of patterns in our *TP53* mutations series, was analyzed. The results showed that 42.8% of C > T preferred C preceded by T (i.e., 5′-TC-3′) [the mutated base is underlined] and 28.6% of the C > T preferred C preceded by C (i.e., 5′-CC-3′) (supplementary Table S2). This result suggests the possible involvement of APOBEC enzymes in *TP53* mutations in Rwandan gastric cancer.

## Discussion

In the present study, a *TP53* mutation was observed in 24 (25.3%) of the 95 Rwandan patients with gastric cancer, and a total of 29 *TP53* mutations were identified. These *TP53* mutations were distributed between exon 4 and 8, and a majority of these mutations (65.5%) were missense mutations. Immunohistochemical analysis for *TP53* showed a TP53 protein accumulation in most of *TP53* missense mutation-positive cases. Among 29 *TP53* mutations, one was novel (c.459_477delCGGCACCCGCGTCCGCGCC) and this 19-bp deletion mutation in exon 5 caused the production of truncated TP53 protein (p.G154Wfs*10). Regarding the spectrum of *TP53* mutation, G:C > A:T at CpG sites was the most prevalent and G:C > T:A was the second most prevalent. Interestingly, when the spectrum of *TP53* mutations was compared between our study and three previous *TP53* mutational studies for non-Rwandan patients with gastric cancer, G:C > T:A mutations were significantly more frequent in our study than in the three previous *TP53* mutational studies, and even after correction for FDR, statistical significance was observed. These findings suggested that the *TP53* G:C > T:A transversion mutation in Rwandan patients with gastric cancer was more frequent than in non-Rwandan patients with gastric cancer, probably due to a different etiological and carcinogenic process of gastric cancer in Rwanda. This report is the first published study to describe the gene mutation in Rwandan patients with gastric cancer, providing an important genetic analysis of Rwandan gastric cancer.

In this study, the percentage of gastric cancer cases with *TP53* mutations was 25.3%, this result is comparable with the incidence of mutation (27.0%) in the previous study by Li-Chang et al. [54], slightly lower when compared to the findings of Hwang et al., 37.4% [55] and 43.3% of Tahara et al. [56]. This difference could partly be due to the relatively small sample size of this study compared to those studies with 110 to 2946 gastric biopsies. However, there are other studies with fewer mutations but relatively big sample sizes. In a study done in India, the frequency of *TP53* mutations in 348 gastric cancer biopsies was 4.6% [57], in Latin America even though the sample size was small, the number of mutations was still low at 3.5% in 59 gastric cancer biopsies [58]. With these findings, one cannot be confident in asserting that the *TP53* gene is less mutated in Rwandan cancer or in Africa compared to Europe, Latin America or Asia since studies done on the African continent analyzing *TP53* mutations in gastric cancer are still lacking [24, 59]. Different reports suggest that differences in patients’ constitution, methods employed in the detection of mutations, or the anatomical sites of the tumor can result in differences in the prevalence of *TP53* mutations [60]. For instance, in a study conducted by Tolbert et al., *TP53* mutations were found in 54% of tumors in the cardia versus 25% of tumors in the antrum [61].

In our study, we found a significantly higher occurrence of the G:C > T:A transition in the TP53 mutation pattern in gastric cancer patients from Rwanda compared to those from non-African countries such as China, Hungary, Japan, Poland, Romania, the USA, and Hong Kong [33, 43, 52]. The distribution of G:C > T:A in Rwandan and non-Rwandan patients with gastric cancer did not show any statistically significant difference in terms of age and sex (supplementary Table S5). Due to the fact that the gastric cancer sample in this study was obtained through endoscopic examination, it is difficult to compare the histopathological classification and staging of cancer between the Rwandan patient group and the non-Rwandan patient group. It is possible that regional differences are associated with the variation in the frequency of G:C > T:A transition. The variation in environmental factors, infectious diseases, food contamination, and socio-economic status between Rwanda and other countries may result in a unique mutation spectrum specific to Rwanda. This mutation pattern with G > T as the main feature has been previously associated with liver and lung cancers based on the mutagen [12, 13]. Liver cancer associated with the dietary mutagen aflatoxin ranks fifth with a prevalence rate of 4.22/100,000 in Rwanda [14]. Aflatoxin exposure is associated with aflatoxin B1 (AFB1)-N7-guanine adducts in HCC [62], and a unique transversion at codon 249 (p.R249S; G:C > T:A) is highly prevalent in areas where aflatoxin is a common food contaminant [12]. Surprisingly, in our study, this particular transversion was not identified in any cases with G:C > T:A; instead, the most prevalent transversion was at codon 176 (c.527G>T), which appeared 3 times. Aflatoxin contamination results from poor food storage and inadequate drying facilities and is a major issue in Rwanda and Africa in general [63, 64]. Conversely, aflatoxin has not been considered a public health concern in the United States and other developed nations. Nevertheless, the growth and production of aflatoxins by toxigenic fungi may also change in distribution with global climate change [65, 66]. Studies to assess the epidemiological impact of aflatoxin in Rwanda and to uncover genomic changes that may be attributable to aflatoxin in Rwandan patients with cancer are necessary. Tobacco smoking-associated lung cancer ranked seventh in Rwanda, with a prevalence rate of 3.16/100,000 [14]. While the burden of smoking-related diseases remained unestablished in Rwanda due to insufficient data [67], 7% of males and < 1% of females were known to use any type of tobacco during 2019–2020 [68]. However in the United States, smoking killed approximately 29.5 million Americans from 1960 to 2020 [69]. Additionally, the leading cause of cancer death is lung cancer in the United States [70] and smoking in Eastern Europe [71, 72]. The smoking-related cancer mortality rate was 337.2/100,000 among males and 157.3/100,000 among females in China during 2002–2018 [73]. Additionally, 145,765 new cancer cases and 72,520 cancer deaths were attributable to active tobacco smoking in 2015 in Japan [74]. Tobacco smoke induces PAH-N^2^-guanine adducts and is associated with bronchial and lung cancers, head and neck cancers, and esophageal cancer [12, 75, 76]. The increased ratio of G:C > T:A may also reflect an increase in oxidative stress induced by continued inflammation of the gastric mucosa or environmental oxidative mechanisms such as ionizing radiation [33, 77, 78]. In this study, we were not able to determine whether the changes from G > T in Rwandan patients with gastric cancer were due to AFB1 or tobacco smoke-related adducts. Moreover, whether this mutation pattern is a signature of a mutational process in stomach cancer in Rwanda or in Africa in general, research has to be carried out on a larger scale to understand its contribution to the development of gastric cancer.

In our study, we found that G:C > A:T (G > A and C > T) was 48.3% of all mutations. The G:C > A:T transitions at CpG sites were the most common pattern in all mutations with 34.5%, compared to 13.8% of G:C > A:T non-CpG sites. These results show that CpG sites are the preferred locations for G:C > A:T patterns, a finding that is consistent with other studies [13, 33, 56]. The observed transitions at CpG sites are generally attributed to the high mutability of CpG sites as a result of spontaneous oxidative deamination of 5-methylcytosine [79]. This deamination will result in the change from C to T and it is thought to be catalyzed by members of the cytidine deaminase family, which include activation-induced cytidine deaminase (AICDA) and APOBEC. These enzymes show a strong preference for deaminating cytidine residues depending on the nitrogen base that comes before the mutated base. For instance, APOBEC3A, APOBEC3B, APOBEC3C, and APOBEC1 favor C residues flanked by T, whereas APOBEC3G favors C flanked by C [13, 53, 80]. Studies have shown that the flanking sequence context of a mutation (bases that come immediately 5′ and 3′ to the mutated base) are important in defining the mutation process of a particular cancer [9, 11, 13]. The results of our study revealed that 42.8% of C > T preferred C preceded by T (TC) and 28.6% of the same mutation pattern preferred C preceded by C (CC). This suggests that APOBEC enzymes were involved in this mutational change. While these enzymes normally function as DNA modifiers in physiological processes, their extreme activation by endogenous or exogenous factors may cause DNA damage due to mutations that are not corrected [9].

Some authors found that the G:C > A:T pattern that involves cytidine to uracil deamination is implicated in the mutation process likely to be linked with *H. pylori-*associated gastric cancer [56, 80]. It is believed that the change from C to T is induced by nitric oxide, which is also induced by *H. pylori.* In our study, it is difficult to ascertain the contribution of *H. pylori* to this mutation process because of its association with alterations that occur in cytidine preceded by purine (G or A), and the primary enzyme for this is AICDA [80]. As noted earlier in the discussion, the greater percentage of C > T mutations occurred at C residues preceded by T or C. These results minimize the role that *H. pylori* would have played in this process. The pattern from G:C to A:T mutations with G to A transitions are believed to be partly caused by alkylating agents like *N*-methyl-*N’*-nitro-*N*-nitrosoguanidine, and *N*-methyl-*N*-nitrosourea, which introduce a cytotoxic *O*^6^-alkylguanine into the DNA. Once generated in the DNA, during replication, this abnormal base mispairs with thymine instead of cytosine. Thus, the guanine-cytosine pair gets replaced by an adenine-thymine pair in the final sequence [81]. These alkylating substances, which are considered to be carcinogens in gastric cancer, are widespread in the environment and can be found in foods [82, 83]. This supports the theory that the interaction between humans and environment plays a key role in *TP53* mutations [80]. Looking at the bases that precede the mutated base in C > T transitions (T for APOBEC3A, APOBEC3B, APOBEC3C and APOBEC1 or C for APOBEC3G) and considering the role of alkylating agents on G > A transitions, deaminating enzymes of the APOBEC family and alkylating agents are suspected to be the major causes of G:C > A:T mutations in Rwandan patients with gastric carcinoma. In addition to the above factors, genetic background may also influence the mutation spectrum. Thus, it is important to analyze genetically and environmentally diverse populations.

Mutations in this study were distributed between exons 4 and 8, which is common with studies conducted in eastern Europe and Asia [33]. Hainaut found the majority of these mutations were localized in hotspots regions [12]. Some variants occurred more than once in different patients, including rs397516436 (c.637C>T, p.R213*) and rs786202962 (c.527G>T, p.C176F), which appeared three times each, rs28934575 (c.733G>A, p.G245S) and rs28934578 (c.524G>A, p.R175H) appearing twice each. According to Hainaut and Pfeifer, mutation hotspots at codons 175, 213, and 245 are part of six “major hotspot” codons (175, 213, 245, 248, 273, and 282) that each comprise of at least 2% of all *TP53* mutations in the COSMIC database, whereas codon 176 is part of 13 codons that are considered “mini hotspots” (158, 176, 179, 193, 195, 196, 220, 249, 266, 278, 306, 337, and 342), because they comprise between 1% and 2% of all mutations [12].

In this study, 19 (65.5%) missense mutations, 5 (17.2%) nonsense mutations, and 3 (10.3%) were deletion types resulting in a frameshift. These results are consistent with previous studies where missense mutations were predominant [84]. The results of *TP53* missense mutations as confirmed through TP53 immunostaining are consistent with the previous finding where mutant TP53 exhibits a longer half-life than wild-type TP53 [85]. The results suggest that missense mutation can be suspected whenever TP53 immunostaining shows a diffuse and strong nuclear immunoreactivity.

In this study, there were three frameshift mutations (deletions), one of them novel. The results from *in silico* tools show that this produces a truncated protein. Previous findings suggested that these types of mutations may interfere with protein translation and result in the production of an incomplete protein [86]. Studies indicate that TP53 frameshift mutants lack C-terminal sequences and exhibit a mixture of residual antiproliferative (cellular senescence and aging) and neomorphic functions that may be differentially exploited for targeted therapy [87].

In this study, 7.3% of cases had *ureC* gene detected by PCR indicating *H. pylori* colonization. This finding is consistent with a previous study [49]. Usually, *H. pylori* colonizes the nontumor, non-metaplastic mucosa, and is mostly found in acute gastritis and chronic active gastritis [88]. In our case, we were analyzing gastric biopsies composed largely of tumor. In studies where *H. pylori* was detected in nontumor tissues the positivity was higher than 7.3% [32], thus the prevalence of *H. pylori* in our samples might not reflect the true picture of *H. pylori* colonization in Rwandan patients with gastric cancer.

Our study was limited, we did not match tumor and nontumor tissue during *TP53* gene sequencing and, thus, were unable to confirm with certainty whether the *TP53* variants were genetic polymorphisms or somatic mutations. In this study variants with < 1% of population-level MAF in the database of 1000 genomes [20] and ExAC [41] were determined to be a mutation. Since the most frequent somatic mutations of the *TP53* gene, such as p.R175H and p.R282W, were observed in less than 1% of population-level MAF, we considered our criteria sufficient for the detection of mutant variations of the TP53 gene. Nevertheless, the genetic analysis in tumors in understudied populations would give a novel insight on gene-environmental interaction in human carcinogenesis [89, 90].

## Conclusion

We performed the first gene mutation analysis for Rwandan patients with gastric cancer, which revealed that the G:C > T:A mutation pattern of the *TP53* gene in Rwanda was more frequent than in non-Rwandan patients with gastric cancer. This may suggest a different etiological and carcinogenic process of gastric cancer in Rwanda.

### Electronic supplementary material

Below is the link to the electronic supplementary material.

Supplementary Material 1

Supplementary Material 2

## Data Availability

Available from the corresponding authors on reasonable request.
