# Correction: The spectrum of *TP53* mutations in Rwandan patients with gastric cancer

**DOI:** 10.1186/s41021-024-00303-x

**Published:** 2024-04-02

**Authors:** Augustin Nzitakera, Jean Bosco Surwumwe, Ella Larissa Ndoricyimpaye, Schifra Uwamungu, Delphine Uwamariya, Felix Manirakiza, Marie Claire Ndayisaba, Gervais Ntakirutimana, Benoit Seminega, Vincent Dusabejambo, Eric Rutaganda, Placide Kamali, François Ngabonziza, Rei Ishikawa, Belson Rugwizangoga, Yuji Iwashita, Hidetaka Yamada, Kimio Yoshimura, Haruhiko Sugimura, Kazuya Shinmura

**Affiliations:** 1 Department of Tumor Pathology, Hamamatsu University School of Medicine (HUSM), 1-20-1 Handayama, Higashi-Ku, 431-3192 Hamamatsu, Shizuoka Japan; 2 Department of Biomedical Laboratory Sciences, School of Health Sciences, College of Medicine and Health Sciences, University of Rwanda, Kigali, P.O. Box 3286, Rwanda; 3 Department of Pathology, University Teaching Hospital of Kigali, Kigali, P.O. Box 655, Rwanda; 4 Université Catholique de Louvain, Médecine Expérimentale, 1348 Brussels, Belgium; 5 Department of Pharmacology, Sahlgrenska Academy, University of Gothenburg, SE-40530 Gothenburg, Sweden; 6 Department of Pathology, School of Medicine and Pharmacy, College of Medicine and Health Sciences, University of Rwanda, Kigali, P.O. Box 3286, Rwanda; 7 Department of Internal Medicine, University Teaching Hospital of Kigali, Kigali, P.O. Box 655, Rwanda; 8 Department of Health Policy and Management, Keio University School of Medicine, 160-8582 Tokyo, Japan; 9 Sasaki Institute Sasaki Foundation, 2-2 Kanda Surugadai, Chiyoda-Ku, 101-0062 Tokyo, Japan

**Correction to: Genes and Environment (2024) 46:8**

10.1186/s41021-024-00302-y

Following publication of the original article [1], the authors reported an error in the name of the fifteenth author. The original version was “Belson Rugwizangonga”. The correct version should be “Belson Rugwizangoga”. The correct author’s name has been provided in this Correction.

The original article [1] has been corrected.
