# Supplementary material for: The spectrum of TP53 mutations in Rwandan patients with gastric cancer
Source: Genes Environ. 2024 Mar 8;46:8. doi: 10.1186/s41021-024-00302-y (PMC10921722; doi:10.1186/s41021-024-00302-y)
Supplement: Supplementary file 1 — Supplementary Material 1 [file 41021_2024_302_MOESM1_ESM.docx]

**Supplementary Information**

**The spectrum of *TP53* mutations in Rwandan patients with gastric cancer**

**Nzitakera A et al.**

***Submitted to Genes and Environment***

**Supplementary Figure S1. Determination of the *H. pylori* status in gastric cancer tissue of Rwandan patients by PCR for *H. pylori ureC* gene**

**Supplementary Figure S2**. **Exon/intron distribution of *TP53* mutations in Rwandan patients with gastric cancer**

**Supplementary Figure S3. Types of *TP53* mutations in non-Rwandan patients with gastric cancer**

**Supplementary Table S1. Primers for PCR amplification of TP53 gene**

**Supplementary Table S2. List of *TP53* mutations and flanking bases of the mutated residues in Rwandan patients with gastric cancer [Excel sheet]**

**Supplementary Table S3. Relationship between the *TP53* mutation effect and patient attributes in Rwandan patients with gastric cancer [Excel sheet]**

**Supplementary Table S4. Relationship between *TP53* mutation patterns and patient attributes in Rwandan patients with gastric cancer [Excel sheet]**

**Supplementary Table S5. Clinical characteristics of gastric cancer patients with G:C>T:A *TP53* mutation in studies used for comparison [Excel sheet]**

**Supplementary Table S6. Distribution of single base substitution-type *TP53* mutations according to the 96 mutation patterns [Excel sheet]**

**Supplementary Figure S1. Determination of the *H. pylori* status in gastric cancer tissue of Rwandan patients by PCR for *H. pylori ureC* gene.** PCR amplification of 300-bp fragment of *ureC* gene is shown in 4 gastric cancer cases marked with red font and positive control case.

**Supplementary Figure S2**. **Exon/intron distribution of *TP53* mutations in Rwandan patients with gastric cancer.** The Y axis shows the percentage of mutation per exon/intron.

**Supplementary Figure S3**. **Types of *TP53* mutation in non-Rwandan patients with gastric cancer.** These pie graph data are from three previous studies: (1) the study by Natsume *et al*., *n* = 272, (2) TCGA dataset, *n* = 226, and (3) the study by Wang *et* *al*., *n* = 58.

**Supplementary Table S1. Primers for PCR amplification of TP53 gene**

| Exon | Forward primer (5'-3') | Reverse primer (5'-3') | Size (bp) |
| --- | --- | --- | --- |
| 2 | ATGCTGGATCCCCACTTTTC | TCCCACAGGTCTCTGCTAGG | 205 |
| 3 | GCAGAGACCTGTGGGAAGC | AGGGGGACTGTAGATGGGTG | 197 |
| 4 | ACCTGGTCCTCTGACTGCTC | TTGAAGTCTCATGGAAGCCAG | 355 |
| 5 | TTGTGCCCTGACTTTCAACTC | ACCAGCCCTGTCGTCTCTC | 263 |
| 6 | TCAGATAGCGATGGTGAGCAG | GGAGGTCAAATAAGCAGCAGG | 313 |
| 7 | CTCATCTTGGGCCTGTGTTATC | GAAGAAATCGGTAAGAGGTGGG | 231 |
| 8 | CTGCCTCTTGCTTCTCTTTTCC | TCCTCCACCGCTTCTTGTC | 201 |
| 9 | AGGGTGCAGTTATGCCTCAG | ACTTTCCACTTGATAAGAGGTCCC | 165 |
| 10 | CTTCTCCCCCTCCTCTGTTG | AACCTAGGAAGGCAGGGGAG | 182 |
| 11 | TGATGTCATCTCTCCTCCCTG | GAGGCTGTCAGTGGGGACC | 152 |
